# Occurrence of Mycotoxigenic *Fusarium* Species and Competitive Fungi on Preharvest Maize Ear Rot in Poland

**DOI:** 10.3390/toxins11040224

**Published:** 2019-04-15

**Authors:** Karolina Gromadzka, Lidia Błaszczyk, Jerzy Chełkowski, Agnieszka Waśkiewicz

**Affiliations:** 1 Department of Chemistry, Poznań University of Life Sciences, ul. Wojska Polskiego 75, 60-625 Poznań, Poland; agnieszka.waskiewicz@up.poznan.pl; 2 Institute of Plant Genetics, Polish Academy of Sciences, ul. Strzeszyńska 34, 60-479 Poznań, Poland; lgol@igr.poznan.pl (L.B.); jche@igr.poznan.pl (J.C.)

**Keywords:** mycotoxins, maize ear rot, *Trichoderma*, *Sarocladium*, *Lecanicillium*, *Fusarium* species

## Abstract

Maize has become one of the most important crops for food and feed production—both as a silage and crop residue worldwide. The present study aimed to identify the co-occurrence of *Fusarium subglutinans*, *Fusarium verticillioides*, *Trichoderma atroviride*, *Sarocladium zeae,* and *Lecanicillium lecanii* on maize ear rot. Further, the accumulation of mycotoxins as secondary metabolites of *Fusarium* spp. in maize ear samples was also analyzed. Maize ear samples were collected between 2014 and 2017 from two main maize growing areas in Poland (Greater Poland and Silesia region). A significant difference was found in the frequency of two main *Fusarium* spp. that infect maize ears, namely *F. subglutinans* and *F. verticillioides*. In addition to *Fusarium* spp. *T. atroviride*, *S. zeae,* and *L. lecanii* were also identified. *T. atroviride* species was found in 14% of maize samples examined between 2014 and 2017, particularly with a high percentage of *Trichoderma* spp. recorded in 2014, i.e., in 31% of samples. However, mycotoxin content (beauvericin and fumonisins) varied, depending on both the location and year of sampling. The interaction of fungi and insects inhabiting maize ear and kernel is very complex and not yet elucidated. Therefore, further research is required in this area.

## 1. Introduction

In Poland, maize (*Zea mays*) is, one of the major cereal grains, cultivated every year in an area of approximately one million ha. From year to year, its importance in the cultivation is growing [1]. It is also an important continuous breeding progress, which allows for a significant reduction in the thermal needs of maize. Growers provide us with more and more productive varieties suitable for our climatic conditions. The result is a more precise variety matching the climate and geographical region as well as the differentiated direction of use.

An important disadvantage of maize is its susceptibility to *Fusarium* pathogens [2]. They attack plants at different developmental stages, leading to root rot, stalk rot, and ear rot, which are considered the most important disease of crop worldwide [3]. The prevailing pathogen species can vary over the years depending on various factors, such as the continent and region, agroecological conditions [4,5], insect damage [6], other stress factors and susceptibility of cultivars (hybrids) to infection by *Fusarium* species [7]. There are a number of pathways by which *Fusarium* species may infect kernels, resulting in kernel rot or symptomless infection. The incidence of symptomless infection is usually higher than the incidence of kernel rot [8,9]. Kernel infection by any of these fungi can reduce yields and quality, and result in mycotoxin accumulation in grain, such as deoxynivalenol (DON), zearalenone (ZEA), fumonisins (FBs), beauvericin (BEA), enniatins (ENNs), and moniliformin (MON) [7,10,11,12,13].

In Poland, *Fusarium* species occurrence and their mycotoxin production have been studied since the middle of the 20th century. Until the 1990s, the climate of this country has been described as temperate with relatively cold winters and warm summers. In recent years, the climate has become much warmer, with, frequent, day-to-day and year-to-year variability in the weather patterns noted. As a consequence, the occurrence of other species, such as *F. verticillioides* or *F. subglutinans*, began to increase. Despite this, the level of mycotoxins in cereal grain samples collected in Poland was usually lower in comparison to other European countries [14,15,16]. 

The latest research, which was carried out by NUTRIAD (NUTRIAD Mycotoxin Survey, 2017), showed that 100% of the maize kernels from Poland were contaminated with DON, 94% with ZEA and 72% of the samples contained FBs. The average concentrations of mycotoxins were below EU recommendation levels. The ZEA average concentration was 257 μg kg^−1^, which is high, especially for sows and piglets. The results also showed a high average concentration of DON, 1198 μg kg^−1^. The maximum FBs content was 4920 μg kg^−1^. This high concentration is unusual in Poland and may have a significant effect on the health and performance of farm animals, especially swine and horses [17].

Different strategies have been adopted to reduce *Fusarium* mycotoxin contamination in cereals [18]. A more economical and environmentally attractive option is the use of biological control agents (BCAs) which act as natural antagonists to *Fusarium*. 

*Beauveria bassiana*, *Sarocladium zeae*, and *Lecanicillium lecanii* are endophytes of maize and some other plant species and are proved to be entomopathogenic endophytes that are used in the control of pests under field and laboratory conditions [19,20,21]. *Trichoderma* spp. are among the most studied and promising microorganisms used in a biocontrol system [22,23,24]. Species of the genus *Trichoderma* (teleomorph *Hypocrea*) are found in many ecosystems, but the most common and natural habitat of these fungi is soil [23]. 

Therefore, the aim of this study was to investigate the occurrence of *Fusarium* spp., *Trichoderma* spp., *S. zeae,* and *L. lecanii* in maize kernels collected from two localities in Poland (Greater Poland and Silesia region) during four seasons (2014–2017). Additionally, our hypothesis assumed that naturally occurring endophytes exclude pathogens or/and could decrease the toxin concentrations. The obtained results could be a valuable source of information on the possible use of endophytic fungi in biological plant protection against pathogens.

## 2. Results and Discussion

### 2.1. Occurrence of Fusarium, Trichoderma spp., S. zeae, and L. lecanii in Two Maize Producing Localities in Poland

A significant difference was found in the frequency of the two *Fusarium* spp. that commonly infect maize ears, namely *F. subglutinans* and *F. verticillioides*. Samples originating from locality 1 was dominated by *F. subglutinans*—frequency 54.5–77.2% in all the four years of study (Table 1). The frequency of *F. verticillioides* was found to be 45.4% in 2014 and 11.8–18.2% during 2015–2017.

In samples collected from locality 2, the frequency of *F. verticillioides* was significantly higher at 47.7%, 56.0%, 57.1%, and up to 59.4% in years 2014, 2015, 2016, and 2017, respectively. The frequency of *F. subglutinans* in the same locality was 0% in 2016 and 9.3%, 4.0%, and 21.8% in years 2014, 2015, and 2017, respectively. 

In contrast, the frequency of trichothecenes and zearalenone producing species *F. graminearum* and *F. culmorum* in Poland in the years 1985–2014 was below 16%. In addition, *Fusarium poae* was quite frequent (0–45.7%) [11]. The above-mentioned species were found in high frequency in countries with a warmer climate [13]. *Fusarium poae* frequency was 9.2–26% and this species contributes to the accumulation of beauvericin and enniatins [25]. 

In addition to *F. subglutinans* and *F. verticillioides*, the present study identified the presence of *T. atroviride*, *Sarocladium zeae,* and *Lecanicillium lecanii* in the maize samples. *Trichoderma atroviride* species were found in 50 of 369 maize kernels, collected in Poland during 2014–2017 seasons (Table 2). In particular, a high percentage of *Trichoderma* spp. was found in 2014, i.e., in 31 samples. Abundant masses of green spores of *Trichoderma* were found in all maize ears in six samples. In the remaining 46 samples, *Trichoderma* spp. grew together with *Fusarium* spp. on agar plates containing SNA medium. As reported by Jaklitsch [26], *T. atroviride* is mostly found in Europe (Austria and France) and Central and North America, where it is mostly isolated from soil and also found as a contaminant of other *Hypocrea* spp.

In our previous studies in Poland, we identified 14 taxa of *Trichoderma* in various substrates, and *T. harzianum* was the prevalent species [27,28]. *Trichoderma atroviride,* in contrast, accounted for a minor portion of the isolated strains. One of these strains, namely AN35, was isolated from a maize ear sample in 2005. The *T. atroviride* AN35 isolate exhibited antagonistic properties against several toxigenic *Fusarium* spp., and reduced the amount of produced mycotoxins, such as deoxynivalenol and zearalenone, in dual-culture bioassay [29,30,31]. The same isolate was the most efficient producer of 6-PAP under laboratory conditions. 6-PAP inhibited the growth of several *Fusarium* spp. when spiked per plug from 0.1 to 2 μg on PDA medium [32]. This is the second report where *T. atroviride* species was isolated from maize kernels in Poland. Most of the existing literature describes the occurrence of *T. harzianum* in maize seeds [33,34,35].

In representative samples, the species *S. zeae* and *L. lecanii* were identified using DNA assays. However, in eight samples in 2015, only *S. zeae* was found and no *Fusarium* spp. were detected. 

Strains of *L. lecanii* are known components of bioinsecticides, which are produced on a commercial scale mostly in Asia, India, and South America [21]. In previous studies, *S. zeae* and *L. lecanii* were identified as members of *Acremonium strictum* clade (previously *Verticillium* complex). A more detailed description of the above-mentioned two endophytes was obtained after the use of a phylogenetic approach [36]. Because these endophytes have a very slow growth rate, many difficulties were encountered while isolating their representative strains, especially when they were present in the same maize kernels together with *Fusarium* spp. Moreover, it was rather impossible to isolate them when fast-growing *Trichoderma* spp. were also present in the same maize kernels. Both endophytes are difficult to isolate using procedures described in most previous studies. Low-nutrient agar SNA prepared according to Nirenberg [37] was found to be particularly useful to isolate and identify all the above-mentioned fungi species from maize kernels. *Lecanicillium lecanii* produced vertilecanins, and *Acremonium zeae* produced dihydroresorcylides [38,39].

*Fusarium verticillioides* may be a primary causal agent of disease, a secondary invader or an endophyte, and systemically colonizes kernels. The fungus infects the emerging maize seedlings, the maturing plant, and the new kernel. This species is also frequently recovered from healthy maize seeds and has been known for many years to be an endophyte of maize [40].

It has been shown that another endophyte of maize, *A. zeae*, is a producer of antibiotics that inhibit *F. verticillioides* and *Aspergillus flavus* [41]. Recently, isolates of this species were renamed as *Sarocladium zeae* [39,42]. Most recently, a comparison of *Sarocladium* spp. was reported by Yeh and Kirschner [43]. In addition, the authors derived the phylogenetic tree of *Sarocladium* spp., and identified a new endophyte *S. spinificis* occurring in grasses in Taiwan.

The interaction of *F. verticillioides* species with maize plant and with other fungi, including the pathogens *F. graminearum* and *F. poae*, and with endophytes, such as *S. zeae* (formerly *A. zeae*), and hyperparasites, such as *Trichoderma,* is very complex and may influence the final contamination of kernels with fumonisins and other mycotoxins under field conditions.

### 2.2. Mycotoxin Accumulation

The amount of mycotoxin accumulated in maize kernels in the two localities with *F. subglutinans* (*F. temperatum*) and *F. verticillioides* as the prevalent species is given in Table 3. A significantly higher amount of BEA was found in samples from locality 1—Greater Poland—except in the 2016 harvest. The highest amount of BEA content was recorded in 2015 in locality 1 where the maximum and average concentrations were 1731.55 and 201.33 μg g^−1^, respectively. The lowest toxin content occurred in 2017 in both studied locations. In locality 1, despite a large number of positive samples (95.24%), the average toxin content was 32.25 μg g^−1^. In locality 2, the value was even lower and reached an average of 1.84 μg g^−1^, with a maximum value of 24.14 μg g^−1^.

In China, BEA was the predominant toxin in terms of the frequency and concentration in corn. It was found that 82.3% of samples were contaminated by BEA with the levels ranging from 0.04 mg g^−1^ to 1006.56 mg g^−1^ [44]. The occurrence of beauvericin was also investigated in corn kernel samples collected in Croatia [45]. The crop was found to be contaminated with a mean beauvericin content of 393 ng g^−1^ and the highest level of 1864 ng g^−1^.

The amount of fumonisins B_1_, B_2_, and B_3_ depends on both the location and year of sampling. In this regard, particular attention should be given to the amount of fumonisins in the years 2016 and 2017. The year 2016 was characterized by very high FB_1_ content in both localities, reaching up to 248.85 μg g^−1^ on average in locality 2 when the maximum value was 1418.34 μg g^−1^. In 2016, a significant difference in the frequency of fumonisin occurrence was also noted. In locality 1, these toxins were found in approximately 36% of all the tested samples, whereas in locality 2, the percent of positive samples was almost twice high than that in locality 1. In contrast, the year 2017 was characterized by a very low content of fumonisins, especially in locality 1 where the maximum FB_1_ content in the FDK fraction was 0.81 μg g^−1^. The remaining fumonisins—FB_1_ and FB_2_—occurred at the maximum content of 0.094 μg g^−1^ and 0.022 μg g^−1^, respectively. In locality 2, FB_1_, FB_2_, and FB_3_ content in the same year was 10.21, 0.92, and 0.11 μg g^−1^, respectively. 

According to Gromadzka et al. [12], in Poland from 2005 to 2014, kernels naturally infected by *F. verticillioides* and *F. proliferatum* contained (in mg kg^–1^) up to 710 of FB_1_, 209.72 of FB_2_, and 35.72 of FB_3_. In Italy [46], where the climate is much warmer than that in Poland, the mean levels of fumonisin contamination were remarkably high in years 2006–2008, with the highest value of 10.90 mg kg^−1^ and the lowest value of 4.80 mg kg^−1^. The issue of fumonisin contamination is particularly critical in Africa, where maize is the staple food for the human population and is consumed without any processing. In Eastern and Southern Africa, FB_1_ was detected at concentrations ranging from 0.002 to 1.91 mg kg^−1^, while the sum of fumonisin concentrations in the same samples ranged from 0.002 to 2.73 mg kg^−1^ [47]. 

It is difficult to compare the obtained results in Poland with those obtained in other countries because the final amount of mycotoxins accumulated in maize kernels depends on several factors, such as the toxigenic ability of *Fusarium* spp. [13], maize hybrids produced and their susceptibility to MER [7,48], interaction among the *Fusarium* spp. population in the given area—frequently four species were identified in the examined MER samples [11], the population of European corn borer (ECB) and other pests in maize ears before harvest [6,48,49], and the interaction of *Fusarium* spp. with other fungi, including endophytes and competitive species such as *Trichoderma* or *Alternaria*, *Cladosporium*, *Epicoccum*, *Acromoniella,* and *Nigrospora*, [11], which are proved to be present in the same kernels (ears).

**Figure 1:**
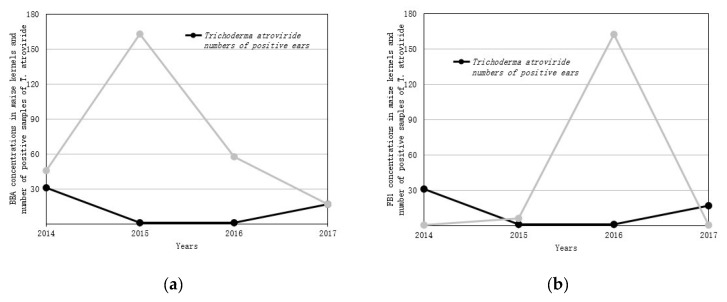
Influence of *Trichoderma atroviride* occurrence on mycotoxins content in maize samples. (**a**) average concentration of BEA, (**b**) average concentrations of fumonisin FB_1_.

The results of our research also indicate the existence of a dependency between the occurrence of endophytic fungi and the content of mycotoxins in the maize kernels. Such trends are noticed only in the case *T. atroviride* (Figure 1). The presence of the other testing endophytic had no effect on toxin concentrations. Our studies were of a preliminary character and should be continued to finally confirm our research hypothesis.

Biological control is a promising strategy for managing MER disease. The use of microbial biological control agents (MBCA) serves as an alternative to chemical control measures for growing pathogen-resistant crop cultivars. *Trichoderma* strains used as biocontrol agents are able to induce plant defense against pathogens and promote plant growth [24,50]. They were also found to reduce mycotoxins, in particular, zearalenone and deoxynivalenol production, in dual-culture bioassay [28,29,30]. Further, the use of microbial biological control agents in agriculture is rapidly increasing because of public concerns about human health, safety of crop products consumed, and impact on the environment. Our studies show that the frequent occurrence of endophytic fungi in our climate zone makes it possible to use them as biological methods of plant protection. In addition, the mycotoxins content decreased as the frequency of endophytes increased. However, further research is necessary to be able to use these fungi in modern agriculture.

## 3. Materials and Methods 

### 3.1. Chemicals and Reagents

Mycotoxin standards (FBs and BEA) HPLC grade solvents and all reagents for extraction and purification process were obtained from Sigma-Aldrich (Steinheim, Germany). Water (HPLC grade) was obtained from MilliQ system (Millipore, Billerica, MA, USA).

### 3.2. Fungal Isolation and Identification

The samples of maize ears were collected in October 2014 (100 samples), 2015 (83 samples), 2016 (58 samples), and 2017 (48 samples) in two main maize growing areas in Poland (locality 1: Greater Poland—16°56′E, 50°58′N and locality 2: Silesia—52°48′N, 16°83′E).

After harvesting, each sample was packed into a separate paper bag and stored at room temperature. The maize ears with *Fusarium* ear rot symptoms were evaluated based on the degree of kernels moldy, shrunken and discolored on a scale of 1% to 100%. Subsequently, small pieces of kernels and visible mycelium from each ear were plated in duplicate on a low nutrient SNA medium and incubated at 20 °C to identify *Fusarium* spp. and other fungi [37,51,52]. The hyphal tips from each culture were placed on both in Petri dishes containing potato dextrose agar and synthetic SNA low-nutrient agar. *Fusarium* spp. were identified according to Kwaśna et al. [52] and Leslie and Summerell [53]. *Trichoderma* spp. were identified according to Kubicek and Harman [54], and *Lecanicillium* and *Sarocladium* were identified according to Shinde et al. [21] and Summerbell et al. [36]. Further, the identification of the selected isolates was performed using DNA assay as described below.

### 3.3. Molecular Identification

For molecular identification, two different phylogenetic markers were selected: the internally transcribed spacer regions 1 (ITS1) and 2 (ITS2,) of the rRNA gene cluster and the fragment (fourth and fifth introns and a portion of sixth exon) of the translation-elongation factor 1-alpha (*tef1*) gene. Fungal isolates were grown in liquid Czapek-Dox broth medium (Sigma-Aldrich, Saint Louis, MI, USA) with the addition of yeast extract (Oxoid™ Yeast Extract Powder Thermo Fisher Scientific, Waltham, MA, USA) and streptomycin sulfate (50 mg L^−1^, AppliChem, Darmstad, Germany) for 21 days on a rotary shaker (120 rpm) at 25 °C. After that, mycelium was collected on filter paper in a Büchner funnel, washed with sterile water, frozen at −20 °C, and freeze-dried. Genomic DNA was isolated using the Wizard^®^ Genomic DNA Purification Kit (Promega, Madison, WI, USA). Amplicons were produced with the primer combination ITS4 and ITS5 [55] for the ITS region and Ef728M [56] and TEF1LLErev [26] for the *tef1* fragment under the following conditions: initial denaturation at 94 °C for 5 min, 35 cycles of denaturation at 94 °C for 45 s, annealing at 58 °C for ITS region, or at 63 °C for the *tef1* gen fragment for 45 s, extension at 72 °C for 1 min, final extension at 72 °C for 10 min. The PCR reaction was carried out in 25 μL reaction volumes with 1 μL 50 ng μL^−1^ of genomic DNA, 2.5 μL 10 × DreamTaq green buffer (includes 20 mM MgCl_2_, Thermo Fisher Scientific, Waltham, MA, USA), 0.2 μL (5 U μL^−1^) DreamTaq green DNA polymerase (Thermo Fisher Scientific, Waltham, MA, USA), 0.2 μL 100 mmol L^−1^ of each primer, 0.25 mM dNTP mix (Sigma-Aldrich, Saint Louis, MI, USA), 19.35 μL sterile distilled water, using a C1000 Thermal Cycler (Bio-Rad, Hercules, CA, USA). Ten μL of PCR product was analyzed on 1.5% agarose gel (BioShop, Burlington, ON, Canada) in 1 × TBE buffer (BioShop, Burlington, ON, Canada) and stained with Midori green advance DNA stain (NIPPON Genetics EUROPE GmbH, Dueren, Germany), visualized under UV light, and photographed (Syngene UV visualizer). Gene Ruler 100-bp Plus DNA Ladder (0,5μg μL^-1^, Thermo Fisher Scientific, Waltham, MA, USA) was used as a size standard. Samples that produced a clear visible band were prepared for sequencing by purifying PCR product (5 μL) with exonuclease I (0.5 μL, 1 U μL^−1^, Thermo Fisher Scientific, Waltham, MA, USA) and shrimp alkaline phosphatase (2 μL, 1 U μL^-1^, Thermo Fisher Scientific, Waltham, MA, USA), incubating 30 min at 37 °C, denaturing 15 min at 80 °C and, cooling down to 4 °C. The 400-bp ITS and 1200-bp *tef1* amplicon purification and sequencing were the same as that described by Błaszczyk et al. [27]. Sequences editing and assembling were performed using the software Chromas v. 1.43 (version 1.43, Technelysium Pty Ltd, Cordelia St, South Brisbane QLD 4101, Australia, 2004). For species identification, the sequences were matched against the nucleotide database using BLASTn (http://blast.ncbi.nlm.nih.gov/). 

### 3.4. Sample Preparation, Extraction and HPLC Analysis 

A detailed procedure of extraction and purification of mycotoxins (FBs and BEA) was reported previously [16,56]. The samples before fumonisins (FB_1_, FB_2_, and FB_3_) analysis were derivatized with *o*-phthalaldehyde (OPA) reagent for 3 min. Methanol: sodium dihydrogen phosphate (0.1 M in water) solution (77:23, *v*/*v*) adjusted to pH 3.35 with phosphoric acid was used as the mobile phase with a flow rate of 0.6 mL·min^−1^. A Waters 2695 apparatus (Waters Division of Millipore, Milford, MA, USA) and a Waters 2475 fluorescence detector (λ_EX_ = 335 nm and λ_EM_ = 440 nm) with a C-18 Nova Pak column (3.9 × 150 mm) were used for fumonisins analysis. HPLC analysis of BEA was performed using a Waters 2695 system equipped with a Waters 2996 Array Detector (at 205 nm) with C-18 Nova Pak column (3.9 × 150 mm). Samples were eluted with acetonitrile: water (70:30, *v*/*v*) at a constant flow of 1 mL min^−1^ for 45 min. The limits of detection were 10 and 8 ng g^−1^ for FBs and BEA, respectively. The obtained positive results (on the basis of retention times) were confirmed by HPLC analysis and compared with the relevant calibration curve (correlation coefficients for FB_1_, FB_2_, FB_3,_ and BEA were 0.9967, 0.9983, 0.9966, 0.9991, respectively). Recovery rates for FB_1_, FB_2_, FB_3_, BEA were 93, 96, 87, and 91%, respectively.

## Figures and Tables

**Table 1:** Frequency of *F. subglutinans* and *F. verticillioides* (in %) in maize ear rot in two localities in Poland during 2014–2017.

Year	Locality 1 (Greater Poland Region)		Locality 2 (Silesia Region)	
	*F. subglutinans*	*F. verticillioides*	*F. subglutinans*	*F. verticillioides*
2014	54.5	45.4	9.3	47.7
2015	60.0	13.3	4.0	56.0
2016	55.8	11.8	0.0	57.1
2017	77.2	18.2	21.8	59.4

**Table 2:** Occurrence of *Trichoderma atroviride*, *Sarocladium zeae,* and *Lecanicillium lecanii* species in maize ears with significant *Fusarium* ear rot in Poland during 2014–2017 seasons.

Year	Numbers of Examined Ears	*Trichoderma* atroviride* Numbers of Positive Ears	*Sarocladium zeae* and Lecanicillium lecanii* Numbers of Positive Ears
2014	100	31	1
2015	83	1	35
2016	58	1	8
2017	48	17	16
total	289	50	60

^*^ Molecular identification of 18 isolates confirmed the following species: *Trichoderma atroviride* in six isolates, *Sarocladium zeae* in seven isolates, and *Lecanicillium lecanii* in five isolates

**Table 3:** Average and maximum content [μg g^−1^] of beauvericin and fumonisins in *Fusarium* contaminated kernels in maize samples collected from two localities (Locality 1—Greater Poland region, Locality 2—Silesia region) in Poland during 2014–2017.

Year		BEA [μg g^−1^]		Fumonisins [μg g^−1^]					
				FB_1_	FB_2_	FB_3_	FB_1_	FB_2_	FB_3_
		Locality 1	Locality 2	Locality 1			Locality 2		
2014	average	61.02	31.20	0.89	0.20	0.02	0.13	0.08	0.005
	maximum	445.43	119.52	31.84	7.24	0.19	1.73	1.19	0.12
	%positive	84.62	50.00	50.00	15.00	25.00	58.70	21.74	19.57
2015	average	201.33	124.10	6.54	0.33	0.08	5.52	0.93	0.13
	maximum	1731.55	258.88	214.39	10.19	2.85	92.34	16.67	2.35
	%positive	94.59	100.00	83.78	56.76	54.05	95.23	95.23	42.86
2016	average	44.21	70.79	75.41	26.91	4.33	248.85	71.93	19.01
	maximum	474.56	130.99	465.00	282.97	72.89	1418.34	660.75	219.88
	%positive	89.29	96.15	35.71	35.71	35.71	69.23	69.23	69.23
2017	average	32.25	1.84	0.22	0.021	0.005	1.39	0.13	0.01
	maximum	140.69	24.14	0.81	0.094	0.022	10.21	0.92	0.11
	%positive	95.24	50.00	57.89	57.89	47.37	53.85	53.85	42.31
